# Rare additional chromosomal abnormalities in acute promyelocytic leukaemia resulting in rapidly fatal disease: report of a case

**DOI:** 10.1002/jha2.349

**Published:** 2021-11-30

**Authors:** Ahmed Maseh Haidary, Sarah Noor, Sahar Noor, Maryam Ahmad, Ahmad Walid Yousufzai, Ramin Saadaat, Zeeshan Ansar Ahmed, Abdul Jamil Rasooli, Ahmad Shekib Zahier, Haider Ali Malakzai, Abdul Sami Ibrahimkhil, Samuel Sharif, Mohammad Sarwar Anwari, Abdul Hadi Saqib, Tawab Baryali, Najla Nasir

**Affiliations:** ^1^ Department of Pathology and Clinical Laboratory French Medical Institute for Mothers and Children (FMIC) Kabul Afghanistan; ^2^ Department of Haemato‐Oncology Ali‐Abad Teaching Hospital Kabul Afghanistan; ^3^ Department of Paediatric Medicine French Medical Institute for Mothers and Children (FMIC) Kabul Afghanistan; ^4^ Department of Haemato‐Oncology Jumhoriat Hospital Kabul Afghanistan; ^5^ Department of Pathology and Laboratory Services Agha Khan University Karachi Pakistan; ^6^ Department of Haemato‐Oncology Amiri Medical Complex Kabul Afghanistan; ^7^ Department of Quality French Medical Institute for Mothers and Children Kabul Afghanistan; ^8^ Department of Internal Medicine Rabia Balkhi Hospital Kabul Afghanistan

**Keywords:** acute promyelocytic leukaemia (APL), additional chromosomal abnormalities (ACA), fatal disease, rare

## Abstract

**Background:**

Acute promyelocytic leukaemia results from reciprocal translocation between the long arms of chromosomes 15 and 17. This translocation leads to the formation of chimeric gene, which is both the diagnostic marker as well as the therapeutic target of the disease. Additional chromosomal abnormalities are randomly encountered either at diagnosis or during therapy. Here, we present a case of acute promyelocytic leukaemia that had a rare cytogenetic profile at diagnosis.

**Case presentation:**

Our patient was a 14‐year‐old boy, who presented with characteristic clinical and morphological features of acute promyelocytic leukaemia. Karyotypic analysis revealed trisomy of chromosome 8 with deletion of 9p in addition to t(15;17). The patient passed away within the first 8 h of presentation while receiving conventional chemotherapy and haemodynamic resuscitation.

**Conclusion:**

Our patient presented with a rare cytogenetic profile and rapidly progressive disease. According to our extensive literature search, this was the first case of acute promyelocytic leukaemia having pathognomonic t(15;17) along with trisomy 8 and 9q deletion.

**ABBREVIATIONS**
ACA: additional chromosomal abnormalities
AML: acute myeloid leukaemia
APL: acute promyelocytic leukaemia
ATRA: Al‐trans retinoic acid
PML: promyelocytic leukaemia
RARA: retinoic acid receptor alpha
RARa: retinoic acid receptor alpha

## 1 INTRODUCTION

Acute myeloid leukaemia (AML) represents the clonal proliferation of immature precursors of myeloid series in bone marrow and peripheral blood [1]. Various pathophysiological mechanisms are involved in the pathogenesis of acute leukaemia [2]. Whatever the mechanism may be, their ultimate effect is to alter the genetic machinery of the neoplastic cells in a way that, on the one hand, there is uncontrolled increase in proliferation of immature cells and, on the other hand, there is arrested maturation [2].

Acute promyelocytic leukaemia (APL) results from reciprocal translocation between chromosomes 15 and 17, t(15;17) (q24;q21) giving rise to chimeric fusion of oncogene; the *promyelocytic leukaemia* (*PML*) gene and cellular differentiation protein; and the *retinoid acid receptor alpha* (*RARA*) gene resulting in *PML‐RARA* fusion gene [3]. Normal *PML* is a tumour suppressor gene that is transcribed to PML tumour suppressor protein in the cells [4]. Similarly, the normal *RARA* gene is transcribed to another transcription factor, the retinoic acid receptor alpha (RARa) protein that plays important role in granulocyte maturation [5]. In APL, the *PML‐RARA* is transcribed to the functionally dead PML‐RARa protein with resultant unchecked proliferation and arrested maturation at promyelocyte stage [5]. All‐trans retinoid acid (ATRA) is the agent that bypasses the RARa and thus results in cellular maturation and therefore is included in all the chemotherapeutic regimens for APL [5]. There are alternative translocations of *RARA* with other genetic loci that present with disease having similar clinical and haematological features but variable response to chemotherapy [1].

Additional chromosomal abnormalities (ACAs) have been reported in APL in association with t(15;17) [6, 7]. The ACAs have been associated with variable clinical presentations, overall impact on disease severity, response to chemotherapy and disease progression [7].

Here, we present a case of APL with trisomy 8 and deletion 9q in association with t(15;17), that presented with rapidly fatal aggressive disease.

## 2 CASE PRESENTATION

A 14‐year‐old boy from Faryab province of Afghanistan, presented with pallor, weakness and lethargy that developed over a span of 2 weeks. The patient had also developed bleeding diathesis, including epistaxis, gum bleeding, petechiae and purpurae in the abdomen, chest, upper and lower limbs, 3 days before presentation to the hospital. On examination, the patient was markedly pale, lethargic but alert and orientated. There were numerous foci of petechia and purpura all over the body and subcutaneous haemorrhage at the site of various previous venesection for blood examinations. There were no lymphadenopathy and per‐abdominal examination, the patient had no organomegaly.

**FIGURE 1:**
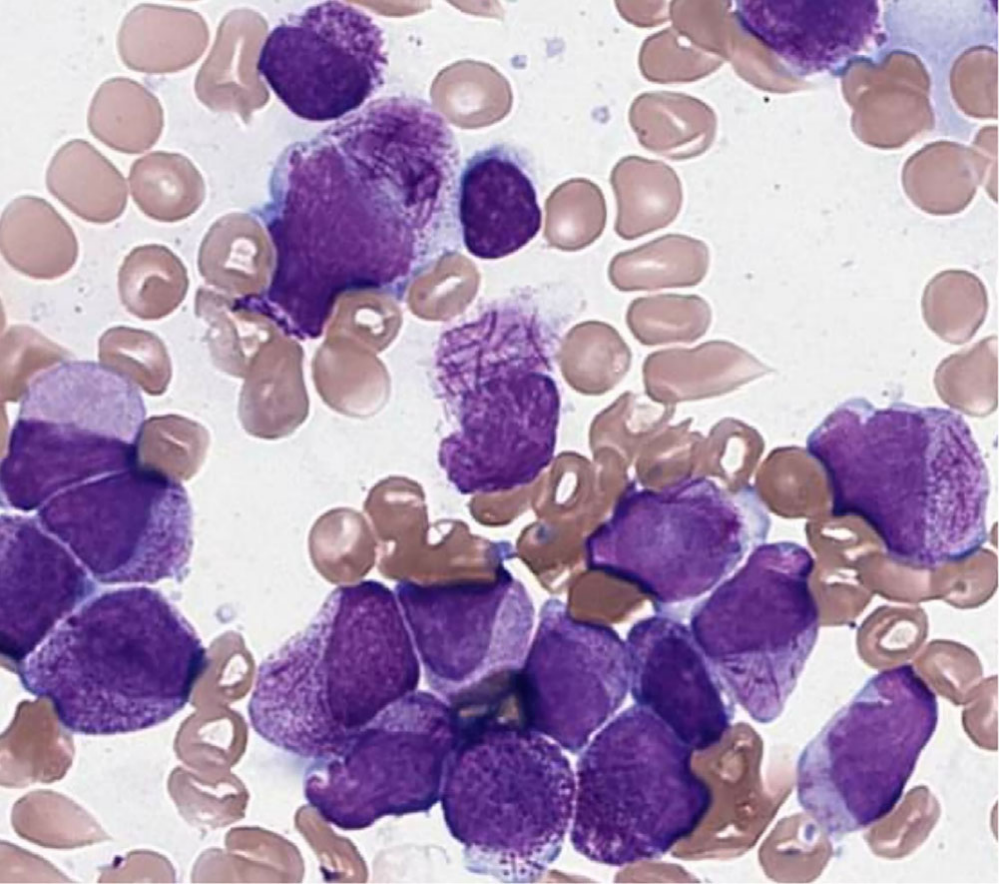
Peripheral blood film stained by Wright‐Giemsa stain demonstrated presence of multiple abnormal promyelocytes with numerous intracytoplasmic Auer rods

Blood examination revealed severe normochromic‐normocytic anaemia with severe thrombocytopaenia, as shown in Table 1. The differential white cell count reported by the analyzer demonstrated erroneous high percentage of neutrophils. Peripheral blood film examination confirmed the diagnosis of APL with presence of 86% abnormal promyelocytes, most of them demonstrating presence of numerous cytoplasmic Auer rods “Faggot cells,” as shown in Figure 1.

**TABLE 1:** Initial laboratory investigations

Laboratory investigations			
Parameters	Values	Parameters	Values
Complete blood count		Biochemistry	
		Liver function test	
Hemoglobin	56 g/L		
Hematocrit	16.3%	AST	28 U/L
Total White cell count	8 × 10^9^/L		
Neutrophil	3%	ALT	18 U/L
Lymphocyte	6%		
Eosinophil	8%	Total bilirubin	1.3 μmol/L
Monocyte	10%		
Promyelocytes	73%	Direct bilirubin	0.6 μmol/L
Platelet	5 × 10^9^/L		
Coagulation profile		Indirect bilirubin	0.7 μmol/L
PT	24 s	Renal function test	
APTT	68 s	BUN	11 μmol/L
		Createnine	2.1 μmol/L

Abbreviations: ALT, alanine transaminase; APTT, activated partial thromboplastin time; AST, aspartate transaminase; BUN, blood urea nitrogen; PT, prothrombin time.

Accordingly, the patient was commenced with prednisolone (1 mg/kg/ day), ATRA, 45 mg/m^2^ of the body per day, and arcenic tri‐oxide (0.15 mg/kg/day) along with haemodynamic resuscitation including packed RBCs, fresh frozen plasma and platelet transfusion.

**FIGURE 2:**
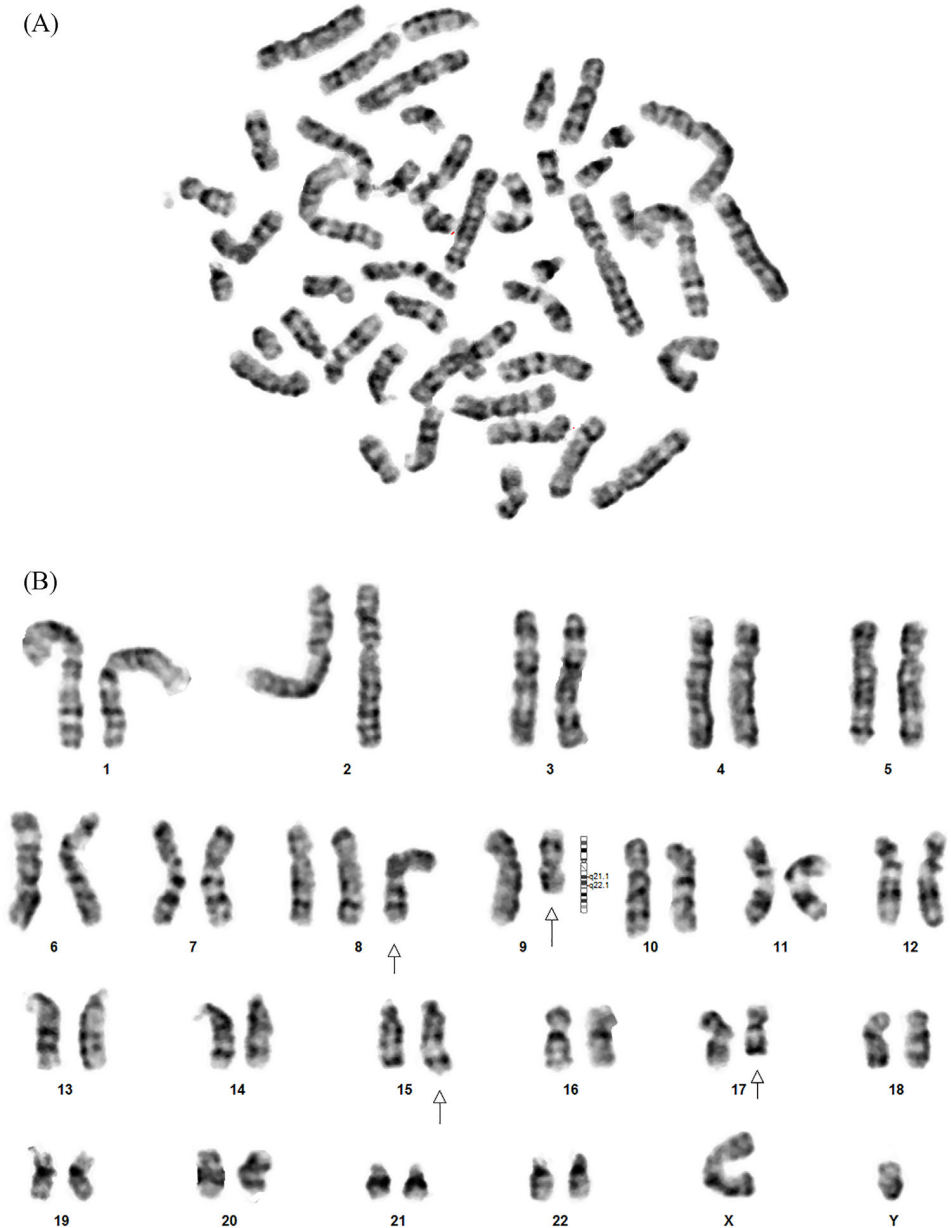
(A) Giemsa‐stained metaphase spread, acquired after 48 h of incubation in culture media. (B) Metaphase spread from a non‐stimulated culture of peripheral blood sample demonstrating 47, XY, +8, del [9] (q21.1q22.1); t (15;17) (q24; q21)

Parents of the patient did not consent for bone marrow examination and therefore blood sample was sent for conventional karyotypic evaluation. Despite the above‐mentioned measures, the patient passed away 8 h after admission. The karyotypic analysis revealed trisomy of chromosome 8 and deletion of 9q in addition to t(15;17), as shown in Figure 2.

## 3 DISCUSSION

The mechanism underlying the pathogenesis of APL is the reciprocal translocation between the long arms of chromosome 15 and 17 resulting in t(15;17) and thus the chimeric oncogene *PML‐RARA* [8]. The *PML‐RARA* chimeric gene and its transcription product, the PML‐RARa protein, are the diagnostic, prognostic as well as therapeutic targets for APL [8]. Therefore, very fairly, the success of current management strategies can be attributed to the incorporation of cytogenetic and molecular genetic modalities for detection of t(15;17) and resultant *PML‐RARA*, at diagnosis but also during the monitoring of disease, transforming it to be a curable disease [9].

Recent efforts and advancements in cytogenetic and molecular genetic studies revealed that more than 90% of APL cases harboured the typical t(15;17) with resultant *PML‐RARA* chimeric gene [9]. It was also demonstrated that APL as a disease represented a common clinical presentation of a wide range of translocation, in which chromosome 17 containing the *RARA* gene was persistently being one of the translocation partners [1, 9]. The so‐called variant translocations do have significant impact on the disease including prognosis and outcome [9]. Such case would require additional therapeutic and monitoring interventions [10].

ACAs, which are karyotypic abnormalities present in addition to the pathognomonic t(15;17), can be encountered in APL patients, either at diagnosis or during therapy [11]. When encountered during therapy, they represent clonal evolution. On the other hand, their presence at diagnosis has not been comprehensively studied, thus requiring further elaboration with advance studies.

The ACA can be classified as numerical and structural chromosomal abnormalities [11]. In the numerical type, the most common abnormalities encountered is trisomy 8, which can either be the sole ACA or associated with other numerical and structural abnormalities [1, 11]. On the other hand, structural chromosomal abnormalities include abnormalities involving 9q, 7q, 1p, 11q, 3q, 20q, 17p and complex three‐way variants translocations involving chromosome 15, 17 and another chromosome with resultant PML‐RARA fusion [11]. Rarely, cases have been reported with clinical and morphological features of APL where the cytogenetic evaluation revealed presence of t(15;17) along with other recurrent genetic abnormalities associated with AML, such as t(8;21) [12].

Our patient presented with rapidly progressive and fatal disease, having typical clinical and morphological features of APL. Chromosomal analysis using conventional karyotypic by Giemsa banding revealed presence of trisomy 8, deletion 9q along with the pathognomonic t(15;17). Such karyotypic profile has never been reported elsewhere in the literature. The fact that the patient succumbed in spite of receiving conventional chemotherapy and haemodynamic resuscitation can be a matter of debate and detailed scrutiny. Thus, further multicenter and multinational studies are required for better understanding of the pathogenesis and pathophysiology of APL at molecular levels. This would enable better understanding of additional chromosomal abnormalities and their prognostic implications.

## 4 CONCLUSION

To the best of our knowledge, our patient was the first case of APL to harbour trisomy 8 and deletion 9 in association with t(15;17).

## AKNOWLEDGEMENT

We would like to extend our very sincere gratitude to Dr. Ahmed Nasir Hanifi and Dr. Jamshid Abdul‐Ghafar, who established the Department of Pathology and Clinical Laboratory at FMIC, Kabul, Afghanistan.

## AUTHOR CONTRIBUTIONS

Ahmed Maseh Haidary, Sarah Noor, Sahar Noor, Abdul Jamil Rasooli and Ramin Sadat conceived the idea. Ahmed Maseh Haidary, Zeeshan Ansar Ahmed and Sarah Noor were the major contributor to the writing of the manuscript. Maryam Ahmad, Mohammad Sarwar Anwari, Ahmad Shekib Zahier, Abdul Hadi Saqib and Samuel Sharif collected the laboratory data via integrated laboratory management system (ILMS). Ahmed Maseh Haidary, Ahmad Walid Yousufzai and Sarah Noor diagnosed the case. Sahar Noor and Abdul Jamil Rasooli provided the clinical information of the patient. Abdul Hadi Saqib, Maryam Ahmad and Samuel Sharif performed cytogenetic studies. Sarah Noor, Ahmed Maseh Haidary, Abdul Sami Ibrahimkhil, Najla Nasir, Ahmad Shekib Zahier and Haider Ali Malakzai were the major contributors for critically revising the manuscript for important intellectual content. Najla Nasir, Sarah Noor and Ahmed Maseh Haidary have given expert opinion and final approval of the version to be published. All authors read and approved the final manuscript.

## CONFLICT OF INTEREST

The authors declare no conflict of interest.
